# A direct-to-drive neural data acquisition system

**DOI:** 10.3389/fncir.2015.00046

**Published:** 2015-09-01

**Authors:** Justin P. Kinney, Jacob G. Bernstein, Andrew J. Meyer, Jessica B. Barber, Marti Bolivar, Bryan Newbold, Jorg Scholvin, Caroline Moore-Kochlacs, Christian T. Wentz, Nancy J. Kopell, Edward S. Boyden

**Affiliations:** ^1^Synthetic Neurobiology Laboratory, Media Lab and McGovern Institute, Departments of Brain and Cognitive Sciences and Biological Engineering, Massachusetts Institute of TechnologyCambridge, MA, USA; ^2^LeafLabs, LLCCambridge, MA, USA; ^3^Center for BioDynamics, Department of Mathematics, Boston UniversityBoston, MA, USA

**Keywords:** neural recording, electrode array, FPGA, scalable, data acquisition

## Abstract

Driven by the increasing channel count of neural probes, there is much effort being directed to creating increasingly scalable electrophysiology data acquisition (DAQ) systems. However, all such systems still rely on personal computers for data storage, and thus are limited by the bandwidth and cost of the computers, especially as the scale of recording increases. Here we present a novel architecture in which a digital processor receives data from an analog-to-digital converter, and writes that data directly to hard drives, without the need for a personal computer to serve as an intermediary in the DAQ process. This minimalist architecture may support exceptionally high data throughput, without incurring costs to support unnecessary hardware and overhead associated with personal computers, thus facilitating scaling of electrophysiological recording in the future.

## Introduction

In systems neuroscience there is great interest in recording the *in vivo* activity of ever larger numbers of neurons (Stevenson and Kording, 2011; Alivisatos et al., 2012; Bargmann et al., 2014). Recent progress in microfabrication techniques has led to the development of high-channel count electrode arrays (Einevoll et al., 2012; Ferrea et al., 2012; Maccione et al., 2012; Ballini et al., 2014; Berényi et al., 2014; Ito et al., 2014; Scholvin et al., 2015) for large-scale extracellular recording of neural activity. However, acquiring all of this data is a challenge, which will become more difficult with the ever-increasing scale of recordings. Accordingly, there is a need for a simple, high-performance data acquisition (DAQ) module that can be replicated many times, and organized in parallel, to achieve processing of an arbitrary number of recording channels.

Here we present a novel architecture for a DAQ module in which a digital processor receives data from an analog-to-digital converter, and writes that data directly to hard drives, without the need for a personal computer to serve as an intermediary in the DAQ process. This “direct-to-drive” design is implemented using a field-programmable gate array (FPGA), and employs dedicated data paths on the FPGA for each of the system tasks, yielding guaranteed constant data throughput due to the parallel nature of the circuitry. Each of these modules can acquire data from 1024 neural recording channels at 16-bit depth at 30 kHz, uncompressed.

## Materials and Methods

Software and hardware design files are available for download under the MIT license here: http://www.scalablephysiology.org/willow/. This includes FPGA cores (with the exception of the SATA core), circuit schematics and bills of materials for the FPGA board and power board and interface board, and a custom Python script used to check that no acquired data is missing from storage hard drive after experiments were complete (run-until-failure.py). We also provide a link to our custom GUI software and a User Guide with example data files along with Python and MATLAB scripts to read the data files. From Python or MATLAB, with some additional work, the data can be re-formatted and exported as desired to facilitate cross-platform work flows. Furthermore, to access general purpose input/output (GPIO) data we posted on the website an example data file in which the state of one input GPIO pin is modulated, a MATLAB script to read the data, and plots of the example data.

### Module Design

**Figure 1:**
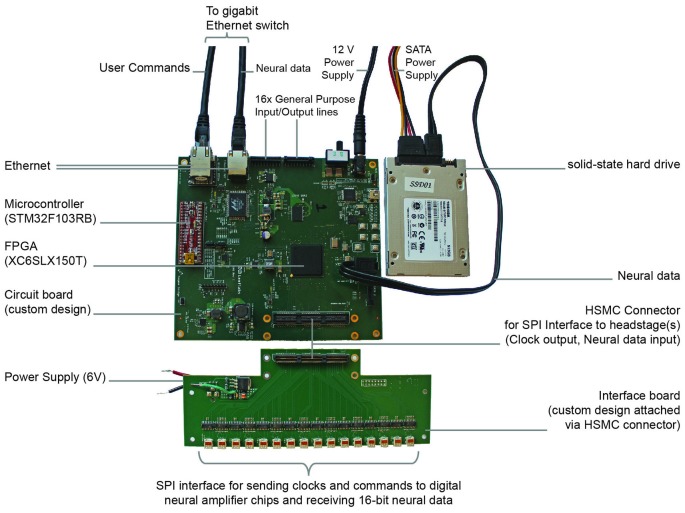
**Implementation of direct communication design of data acquisition (DAQ) module.** Photograph of the acquisition module with key parts labeled. The field-programmable gate array (FPGA)-board measures 15 cm × 16 cm × 2 cm.

We built a minimalist DAQ module (Figure 1) centered around an FPGA (Spartan-6 LX150T, Xilinx, Inc., San Jose, CA, USA) that acquires neural data directly from analog-to-digital converters downstream from amplifiers (“headstages”).

Each of these modules can acquire data from 1024 channels at 16-bit depth at 30 kHz. The module stores data directly to an attached SATA storage device and simultaneously distributes a copy of the neural data over a high speed (1 Gb/s) Ethernet wired network, e.g., to a visualization computer for real-time inspection of neural data. The distributed copy of the neural data consists of either snapshots of all 1024 channels or a continuous stream of one or more selected channels. The module is controlled remotely by sending basic user commands, e.g., start or stop recording, over the network from the visualization computer. In order to avoid collision with streaming neural data, these user commands are handled by a separate, low data rate connection to the module.

In addition to neural data, the module can store experiment protocol information or experimental data, e.g., triggers from beam breaks or for laser pulses, accessible by the experimenter and useful for coordinating overall system operation. To assist in reconstructing a complete neural recording data set from multiple modules being used in the same acquisition session, each module tags neural data with meta-data such as hardware identifiers and time stamps. The total data rate to acquire 1024 channels of data, including auxiliary data, meta-data and zero padding (each described in detail below), is 122.88 MB/s. At this data rate, a 512 GB hard drive, for example, can store up to 70 min of data from 1024 channels.

### FPGA Architecture

**Figure 2:**
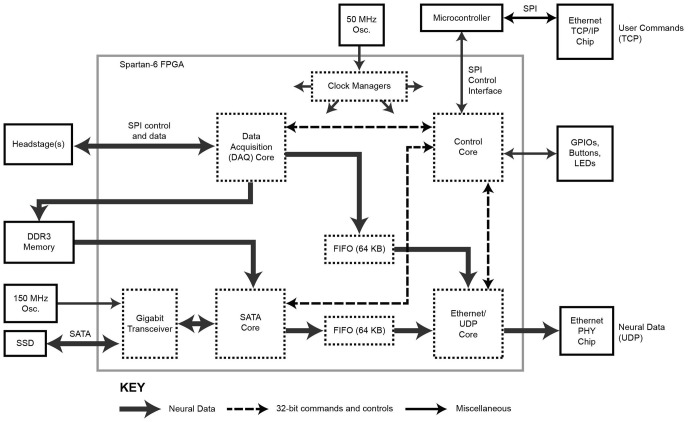
**Block diagram of the FPGA circuitry.** The programmable FPGA circuitry implements a DAQ core to interface with headstages and add meta-data, a SATA core for direct-to-drive data storage, an Ethernet/UDP core for high-speed data transmission over Ethernet, and a Control core containing of a bank of registers for controlling the system. One gigabyte of DDR3 memory buffers acquired data before it is stored to hard drive. The TCP protocol is implemented outside of the FPGA using a separate microcontroller.

The FPGA core architecture is shown in Figure 2. With the exception of the SATA core (IntelliProp, Longmont, CO, USA), all cores were written from scratch and are available for download from the website. To transfer data onto and off the FPGA, we took advantage of built-in, high-speed, serial transceivers, and implemented the industry standard SATA 1 interface (187.5 MB/s) in the FPGA itself as a SATA core. This allows the module to store data directly to an attached SATA storage device at data rate sufficient to capture 1024 neural recording channels, but higher speeds are possible (e.g., by using SATA II or III interface). This core was purchased as closed source and cannot be shared, except as a pre-compiled bitfile.

In addition, a copy of the neural data can be distributed over a network for relay to a visualization computer. To achieve 1 Gb/s data rates over Ethernet, neural data is encapsulated as UDP packets by the Ethernet/UDP core. This core interfaces with an onboard Ethernet controller (Marvell MV88E1111, which implements an Ethernet PHY layer).

The 1024 channels of data are here brought on board using 32 front-end neural amplifier digital-output chips (Intan Technologies Inc., RHD2132). Each chip is capable of 32 channels of amplification and digitization, and the individual SPI signals from each chip are aggregated on a simple interface board (shown in Figure 1). The DAQ core (Figure 2) includes drivers to communicate over a low-level SPI bus protocol to individual chips (according to the RHD2132 specification sheet), higher-level drivers to initialize the chips and acquire data, and an aggregation layer to combine data from all of the chips and make this aggregate data available to other cores through a RAM interface. Details of the SPI communication for the Intan chips we used in this implementation are found in the data sheet.^1^ In addition to sampling the 32 input channels of each chip, the core allows access to a digital-to-analog waveform generation circuit (useful for electrode impedance measurements), auxiliary analog inputs, and the full set of SPI controllable features of the Intan chip.

The Ethernet/UDP, DAQ and SATA cores are governed by a Control core which mediates data flow timing and provides a unified configuration interface. This Control core exposes configuration and control registers over TCP/IP to the network with the help of a simple off-the-shelf, on-board, microcontroller board (Maple Mini, LeafLabs, Cambridge, MA, USA). Offloading the TCP/IP implementation simplifies the FPGA firmware and frees up valuable FPGA resources to be used for other data processing tasks or could allow for the use of a smaller/cheaper FPGA.

In contrast to the TCP protocol, under which the sender keeps a copy of each packet to ensure reliable communication, in the UDP protocol no copy is kept, so delivery of a packet to a user-controlled software application is not guaranteed by the UDP protocol operating alone. Thus, commands sent as TCP packets to the Control core are always delivered, but neural data sent as UDP packets to real-time visualization devices on the network may not. Regardless, a complete record of all acquired data is stored on attached hard drive(s) for full offline analysis.

### Binary Format of Acquired Data

The FPGA is able to acquire data from up to 82 SPI interfaces simultaneously, each running with data rates of 400 Mbits per second. In our implementation, each of 32 headstage circuits (RDH2132) supply 16.8 Mbits of data per second, consisting of both the 32 input channels as well as three auxiliary channels which can be chosen arbitrarily from any of the various registers in the RHD2132 chip, or from the current state of 16 GPIO channels on the DAQ module. By default the FPGA is programmed to automatically record the state of all GPIO pins at 2 kHz sample rate. In aggregate, this amounts to 1120 channels at 30 kS/s and 16-bit resolution.

To these 1120 16-bit neural and auxiliary samples, extra meta-data is prepended to assist in reconstructing a complete data set, i.e., all data collected in the same acquisition session. The meta-data identifies the acquisition session, the recording module identity, the sequence in which the samples were acquired, and which neural channel contributed to the recording. In our implementation, this meta-data amounts to 0.9% of the channel data rate and includes an 8-byte experiment identifier, a 4-byte module identifier, a 4-byte sample index, and a 4-byte chip live status, each described below. First, the experiment identifier is a unique tag shared by all data collected in a given acquisition session; we use a 64-bit UNIX UTC time stamp as the experiment identifier which roughly (but need not exactly) corresponds to the start of acquisition. Second, the module identifier should globally and uniquely identify the module; we use the bottom 32-bits of one of the module’s Ethernet MAC addresses as this identifier. Third, the 32-bit sample index is a sequential sample counter that will roll over to zero after approximately 39 h of experiments when acquiring at 30 kS/s. Fourth, the chip live status value indicates which of the possible 32 Intan chips are active, and thus whether the data corresponding to that chip’s channels should be ignored or analyzed. The combination of 20 bytes of meta-data, 2048 bytes of neural data, and 192 bytes of auxiliary data define a complete module sample. Sets of 128 complete module samples are collected and written as binary data to an attached hard drive.

The purpose of the hard drive is solely to store the data as a single stream. Therefore, a random-access file system is not required, and instead the data is written in a single sequential manner. This helps to improve read/write performance and storage efficiency. When sending data to the hard drive, currently the data is padded with zeros at the end. This padding amounts to 44.8% of the channel data rate and was initially put into place to maintain constant write speeds. However, with the addition to the module of extra memory (e.g., 1 GB DDR3 RAM), this zero padding is not necessary, and could instead be used to store more data in future implementations (up to an additional 831 neural channels and associated auxiliary channels and meta-data, in this implementation).

### Neural Recording

All procedures were in accordance with the National Institute for Laboratory Animal Research Guide for the Care and Use of Laboratory Animals and approved by the Massachusetts Institute of Technology Committee on Animal Care. Mice (C57BL/6, 8–12 weeks old, male, Jax) were placed under general anesthesia, using a rodent anesthesia machine with 0.5–2% isoflurane in pure oxygen. All experiments were performed inside of a grounded Faraday cage to minimize electromagnetic interference. The electrical noise of the complete, powered-on system was measured as 3.9 μV RMS input-referred (600–9700 Hz), including FPGA board, headstages, and custom silicon probes bearing close-packed electrodes (Scholvin et al., 2015), characterized by submerging the array and reference wire and ground wire in 0.9% saline. Then, a 200 micron diameter craniotomy was opened in the skull of an anesthetized mouse, using the autocraniotomy robot we developed in order to perform craniotomies under closed-loop conductance measurement so that brain damage does not occur (Pak et al., 2015). Afterwards, silicon probes were inserted acutely into the brain under computer control (Z812B motorized actuator, TDC001 controller, ThorLabs, Newton, New Jersey; 9066-COM, Newport Corporation, Franklin, MA, USA).

## Results

To validate the proper functioning of the DAQ system, we connected 32 Intan neural amplifier chips (Intan RHD2132), in the form of 8 headstages with 4 Intan chips each, to our custom DAQ module (Figure 1) as input, and a 512 GB solid-state drive (THNSNC512GBSJ, Toshiba, Tokyo, Japan) as output. The module measures 15 cm × 16 cm × 2 cm in size, weighs 164 grams, and dissipates 7 W in full operation. These figures are comparable to the state of the art, e.g., Intan Eval Board, which measures 10 cm × 15 cm × 2 cm, weighs 123 grams, and dissipates 3.5 W. Both Ethernet ports on the module were connected by CAT-5 cables to a network switch (Cisco Catalyst 3560X) which was also connected to a visualization workstation (Intel Core i7 64-bit Dual-Quad Core 2.8 GHz, 8 GB RAM, Ubuntu 14.04 LTS) running custom daemon code written in C and custom GUI code written in Python. Although no personal computer is required for each data storage node to operate, it is helpful to have one computer for user interaction and data visualization.

From the GUI we initiated both a data capture to hard drive of all 1024 channels and at the same time a full sample stream of a single channel. The system ran until the hard drive was full which took about 70 min. Using a custom Python script (see “Materials and Methods” Section) we verified that no error conditions took place (apart from the disk being full), and thus no data loss.

We then disconnected the hard drive from the module and connected it directly to the workstation using a SATA to USB docking station. We downloaded the complete contents of the drive to the workstation for further analysis. Again using the custom Python script we confirmed for each complete module sample that 1024 channels were captured, and that the meta-data was correct (e.g., the hardware and experiment identifiers did not change and that the sample index incremented monotonically without skipping any values). In addition, we verified that the amount of data corresponded to the capacity of the hard drive (e.g., 125026304 complete module samples for the 512 GB drive). No errors were encountered on any of three repetitions of this validation.

**Figure 3:**
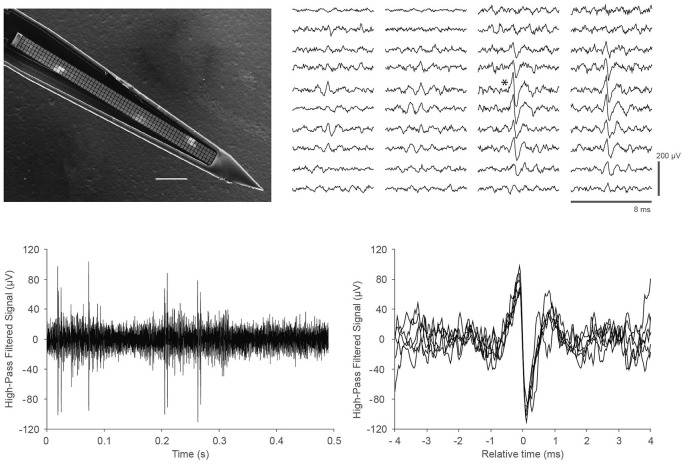
***In vivo* recordings with a close-packed electrode array in somatosensory cortex of a head-fixed mouse under light (0.5%) isoflurane anesthesia. (Top left)** Scanning electron micrograph of a single shank array with an array of 4 × 64 recording sites, visible as light gray squares along the center line of the shank. The shank is 15 μm thick, the recording sites are 9 × 9 μm in size, and spaced at a pitch of 11.5 μm; the 64 rows span a length of 734 μm. Scale bar, 100 μm. Details of the design and fabrication are described in Scholvin et al. (2015). **(Top right)** Example of the recorded data for a single spike across 40 electrodes (4 columns of 10 rows) on a 256-channel probe. Our close-packed design enables the spike to be picked up by several nearby recording sites, facilitating data analysis. The vertical scale bar is 200 μV. **(Bottom left)** Data from a single recording site (marked with an asterisk next to the corresponding trace in the **Top right** panel); **(Bottom right)** Seven spikes from the trace in the lower left are overlaid to show the spike shapes in more detail.

Finally, we demonstrated the ability of the DAQ system to support recording *in vivo* neural activity from somatosensory cortex in mice. We utilized a custom probe with closely packed, spatially oversampling electrode arrays (Scholvin et al., 2015). An example of the data is shown in Figure 3. We recorded similar data for a total of 6.5 h in nine experiments from nine mice.

## Discussion

Existing DAQ systems commonly rely on conventional desktop or server machines for data storage. This works well for small numbers of neural DAQ channels, but at higher channel counts a direct communication design based on FGPAs, such has been implemented for massive DAQ projects in high energy physics (Fischer et al., 2002) and astronomy (Parsons et al., 2008), offers several advantages. Writing data directly to hard drive yields a cost savings, as well as a reduction in complexity. Unlike a CPU, on the FPGA each of the data pathways (connecting sensors, hard drive, and Ethernet) are processed in parallel on dedicated circuitry and have no effect on each other’s performance. With no operating system adding a source of complexity and non-determinism, basic performance guarantees of an FPGA are straightforward and do not require sophisticated techniques such as real time kernels. Furthermore, an FPGA, once programmed, employs dedicated logic for each of the system tasks such as acquiring and storing data, yielding exceptionally high data throughput due to the intrinsically parallel structure of how data streams can flow. Of course, this architecture could also be implemented in an application specific integrated circuit (ASIC), but FPGAs are off-the-shelf parts, reducing entry costs to create such devices, while also allowing for easy modification of the firmware and implemented circuits.

The physical attributes of our system compare favorably to other commercially available systems. Our parts cost was $20 per channel for 1024 channels, with an asymptotic cost of $4 per channel as the number of channels goes up to high channel counts. The retail costs of acquisition systems from Intan^2^ and OpenEphys^3^ range from $50–60 per channel, for 256 channel systems. We note that it is difficult to compare parts costs to retail costs. Not having to rely on the use of personal computers (four per 1024 channels) to do the basic management of the system may be expected to save $4 dollars per channel (assuming $1000 per computer) as the number of channels goes to extremely high numerical values. On the website (see “Materials and Methods” Section) we provide bills of materials and design files for accurate up-to-date cost estimation. Spacewise, a single module and attached hard drive can capture 1024 channels. In comparison, Intan and OpenEphys systems utilize one acquisition module and a computer to capture 256 channels. Thus, for 1024 channels we save the space of four computers minus four hard drives. Finally, the noise of our system (3.9 μV rms) is determined exclusively by the electrode array, printed circuit board (PCB), and headstages. Our headstages were built with Intan neural amplifier chips which have an input-referred noise of 2.4 μV rms according to the datasheet. Thus, our electrode array, PCB, and headstages add approximately 3 μV rms of noise. Like Intan and OpenEphys, our DAQ module is designed to interface using the SPI protocol with digital Intan neural amplifiers. Though we have not tested our module with any headstage other than our own custom 4-Intan chip design, we expect compatibility with other headstages composed of Intan chips, perhaps facilitated by adaptors to match wires appropriately. Design documents describing the SPI interface on the DAQ module are available on the website (see “Materials and Methods” Section).

The implementation described in this paper uses 62% of the available resources of the FPGA chosen (Table 1). With the unutilized resources it may be possible to add further functionality such as real-time lossless compression, which can enable both a higher data transmission density and an increased storage capacity (and thus improved utilization of the hard drives). In principle the duration of recording by the data acquisition system could be made longer by increasing the size and/or number of hard drives. Multiple independent SATA controllers could be embedded in the FPGA firmware to support multiple attached hard drives (for instance, our implementation allows two attached hard drives), allowing for increased recording times or for preventing data loss after a drive failure via redundancy. Furthermore, upgrading the embedded controller to operate at SATA II for faster data write speeds (i.e., 3 Gb/s) to, for example, accommodate more than 1024 channels of neural data, would be straightforward. As noted earlier, the zero padding is not needed thanks to the use of RAM and could be in principle replaced with additional data. In addition, extremely low latency, closed loop control experiments are feasible if control logic is implemented within the FPGA (Hafizovic et al., 2007). Furthermore, it is conceivable that analysis could be performed on the FPGA, with the level of difficulty depending on the complexity of the algorithm. Notably, spike sorting on the FPGA, and only storing the resultant spike information, could in principle yield a dramatic reduction in the rate of output data by several orders of magnitude (Fischer et al., 2002; Schwarz et al., 2014). Since spike sorting is an active area of research (Einevoll et al., 2012), with no universal agreement on the best algorithm, we focus here on capturing the raw neural data. Nevertheless we hope that the FPGA architecture here proposed will provide a platform for the types of analyses needed in spike sorting as they are elucidated.

**Table 1:** **FPGA resource utilization for Xilinx Spartan-6 LX150T**.

Resource	Available	SATA	GigE/UDP	DAQ	Total	Utilization
Slice registers	93980	1667	590	3980	6237	6.64%
Slice LUTs	46648	2569	714	25303	28586	61.28%
BRAM (18 Kbit)	172	3	0	2	5	2.91%
DCM	12	1	0	1	2	16.67%

**Figure 4:**
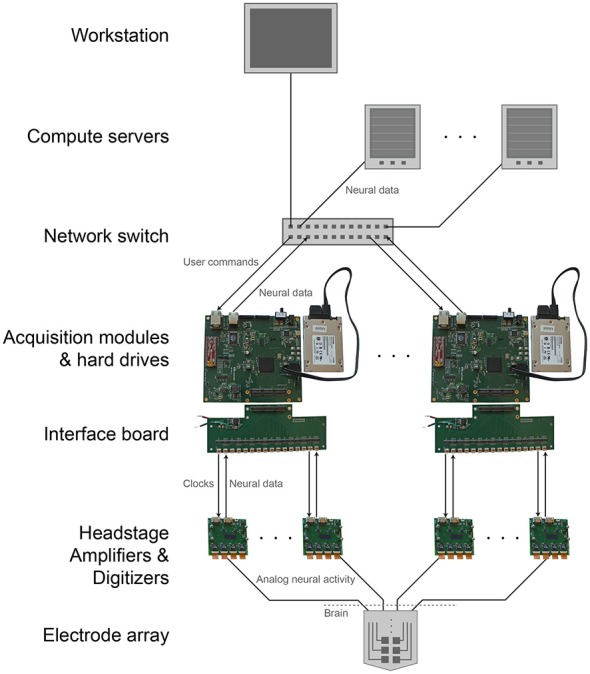
**Scalable DAQ system.** Neural activity is detected by an array of electrodes and conducted over wires to headstages where it is amplified and digitized. An FPGA-based acquisition module communicates with the headstages to receive the neural data and then write it directly to a hard drive. In parallel, over a network the module also transmits the data to a workstation for online visualization and to computer servers for offline analysis. The module receives user commands over the same network.

The modular, parallel architecture will enable scaling by unit increments of 1024 channels, to an arbitrarily large number (Figure 4). This parallelism splits up the original data, and the data can if desired later be recombined on the analysis side, during readout from the individual units. This requires synchronization across individual DAQ modules. A simple yet effective solution for this would be to feed a sample-start pulse from a single source to all of the modules via the GPIO channels at the beginning of every sample acquisition (i.e., at 30 kHz). All of the modules will delay acquisition until this pulse is received, then continuously acquire until halted. This synchronous sampling scheme could be integrated into the existing DAQ FPGA core (with minimal changes) to achieve synchronization well within a sample period (e.g., a microsecond), sufficient for biological recording. Modules would begin acquiring at the same time and would continue sampling at a common rate regardless of individual clock drift. Visualization could take place on multiple computers if desired, each receiving a number of data streams from several modules. Finally, such architectures may be useful for acquisition of other kinds of streaming data of interest in neuroscience, including high-speed neural activity imaging data (Prevedel et al., 2014), or 3-D super-resolution imaging data acquired at high speeds (Chen et al., 2015).

## Conflict of Interest Statement

AJM, JBB, MB, and BN have an equity stake in, and JPK is financially compensated by, LeafLabs, which is selling these data acquisition modules commercially. All other authors declare that the research was conducted in the absence of any commercial or financial relationships that could be construed as a potential conflict of interest.

## Footnotes

^1^http://www.intantech.com

^2^http://www.intantech.com

^3^http://www.open-ephys.org
